# Peginterferon lambda for the treatment of hospitalized patients with mild COVID-19: A pilot phase 2 randomized placebo-controlled trial

**DOI:** 10.3389/fmed.2023.1095828

**Published:** 2023-02-24

**Authors:** Myung-Ho Kim, Josh Elbaz, Nikolaus Jilg, Jenna L. Gustafson, Min Xu, Dilara Hatipoglu, Eric Nohelty, Arthur Y. Kim, Raymond T. Chung

**Affiliations:** ^1^Liver Center, Gastrointestinal Division, Massachusetts General Hospital, Harvard Medical School, Boston, MA, United States; ^2^Department of Internal Korean Medicine, Woosuk University Medical Center, Jeonju, Republic of Korea; ^3^Department of Medicine, Donald and Barbara Zucker School of Medicine at Hofstra/Northwell, Hempstead, NY, United States; ^4^Division of Infectious Disease, Massachusetts General Hospital, Harvard Medical School Boston, Boston, MA, United States; ^5^Department of Medicine, Massachusetts General Hospital, Boston, MA, United States

**Keywords:** COVID-19, peginterferon lambda, SARS-CoV-2 viral load, IP-10 (CXCL-10), CXCL9, (C-X-C motif) ligand 9

## Abstract

**Background:**

This study aimed to investigate the efficacy and safety of subcutaneous injection of peginterferon lambda in patients hospitalized with COVID-19.

**Methods:**

In this study (NCT04343976), patients admitted to hospital with COVID-19 confirmed by RT-PCR from nasopharyngeal swab were randomly assigned within 48 h to receive peginterferon lambda or placebo in a 1:1 ratio. Participants were subcutaneously injected with a peginterferon lambda or saline placebo at baseline and day 7 and were followed up until day 14.

**Results:**

We enrolled 14 participants; 6 participants (85.7%) in the peginterferon lambda group and 1 participant (14.3%) in the placebo group were treated with remdesivir prior to enrollment. Fifty percent of participants were SARS-CoV-2 RNA negative at baseline although they tested SARS-CoV-2 RNA positive within 48 h of randomization. Among participants who were SARS-CoV-2 positive at baseline, 2 out of 5 participants (40%) in the peginterferon lambda group became negative at day 14, while 0 out of 2 participants (0%) in the placebo group achieved negativity for SARS-CoV-2 by day 14 (*p* > 0.05). The median change in viral load (log copies per ml) was +1.72 (IQR −2.78 to 3.19) in the placebo group and −2.22 (IQR −3.24 to 0.55) in the peginterferon lambda group at day 14 (*p* = 0.24). Symptomatic changes did not differ between the two groups. Peginterferon lambda was well tolerated with a few treatment-related adverse effects.

**Conclusion:**

Peginterferon lambda appears to accelerate SARS-CoV-2 viral load decline and improve plasma disease progression markers in hospitalized patients with COVID-19.

## 1. Introduction

Coronavirus disease 2019 (COVID-19), caused by severe acute respiratory syndrome coronavirus 2 (SARS-CoV-2), is becoming endemic. While widespread vaccination has effectively reduced COVID-19 cases, therapeutic options to reduce viral load, relieve symptoms, and prevent disease progression for patients with COVID-19 are still needed. As of October 2022, the US Centers for Disease Control and Prevention recommends antivirals (nirmatrelvir/ritonavir, remdesivir, and molnupiravir) and a monoclonal antibody to the spike protein of SARS-CoV-2 (bebtelovimab) to control SARS-CoV-2 infection (1). However, these approaches are not widely available worldwide, include intravenous options that are difficult to administer, and, especially in the case of monoclonal antibodies, may be evaded by viral mutations that render them ineffective.

Interferon responses constitute the major initial defense against SARS-CoV-2 infection. Type I and III interferons induce the expression of interferon-stimulated genes, which have demonstrated broad antiviral effects (2). Type I interferons induce proinflammatory gene expression by selective induction of the transcription factor interferon regulatory factor 1 (IRF-1). However, Type III interferons, known as interferon lambdas, promote an antiviral state similar to type I interferons without inducing IRF-1-related hyperinflammation (3). Furthermore, interferon lambdas interact with distinct receptor complexes whose expression are limited to epithelial cells in the lung, liver, and intestine, resulting in fewer systemic side effects (2). Peginterferon lambda, a long-acting form of interferon lambda-1a, has been assessed in patients with viral hepatitis B and C which has demonstrated antiviral efficacy similar to type I interferon, but with fewer side effects (4, 5).

In this study, we aimed to investigate the efficacy and safety of subcutaneous injection of peginterferon lambda in patients initially hospitalized with COVID-19 who did not require ventilatory support other than nasal cannula. We tested the hypothesis that peginterferon lambda treatment is associated with SARS-CoV-2 viral load decline compared to placebo.

## 2. Methods

### 2.1. Study design and participants

In this randomized single-blind placebo-controlled study, participants were recruited from patients admitted to Massachusetts General Hospital between July 14, 2020 and July 16, 2021. Patients with SARS-CoV-2 infection confirmed by RT-PCR from nasopharyngeal swab within 48 h of randomization were eligible. Vaccinated subjects were excluded after vaccine became available. Patients with respiratory compromise requiring ventilatory support other than low flow (1 to 6 liter per minute) nasal cannula (heated and humidified high flow nasal cannula, mask, bipap or intubation and mechanical ventilation) were excluded. Other major exclusion criteria included pregnancy and pre-existing immunosuppressive or other medical conditions that could be worsened by peginterferon lambda (see Supplementary protocol for complete inclusion and exclusion criteria). Once inclusion criteria were met, and consent obtained, participants were then randomly assigned to receive peginterferon lambda or saline placebo using a 1:1 password-protected electronic spreadsheet with the randomization allocation. The research ethics board of Massachusetts General Hospital approved the study. This study is registered at ClinicalTrials.gov (NCT04343976).

### 2.2. Procedures

Peginterferon lambda (180 mcg in 0.45 ml volume) was provided by Eiger BioPharmaceuticals for use in this study. At baseline, participants assigned to peginterferon lambda received a 180 mcg subcutaneous injection of the study drug, and those assigned to placebo received a 0.45 ml subcutaneous injection of saline. Nasopharyngeal swabs were collected on days 3, 5, 7, and 14. Swab kits and quantitative RT-PCR testing were supplied by a contract research organization, Viroclinics (Rotterdam, Netherlands). On day 7, participants received a second 180 mcg peginterferon lambda injection and participants in the placebo group received a second 0.45 ml saline placebo injection. On day 7 and 14, participants underwent laboratory measurements to confirm complete blood count and liver function test values were within safe parameters. For research purposes, blood samples were collected at baseline, day 7, and day 14 alongside clinical samples. Plasma levels of cytokines and chemokines (interferon alpha, interferon lambda, IP-10, and MIG) were analyzed by the MILLIPLEX® Human Cytokine/Chemokine/Growth Factor Panel A (HCYTA-60K, Merck Millipore, Billerica, MA). Participants completed a daily symptom questionnaire from baseline through day 14 (see Supplementary protocol for the symptom questionnaire form). Symptoms were reported as not at all, a little bit, somewhat, quite a bit, or very much and scored on a scale of 0 to 4, respectively.

### 2.3. Outcomes

The primary outcome was the proportion of participants with undetectable SARS-CoV-2 RNA at day 7 in each group. Secondary outcomes included the proportion of participants with SARS-CoV-2 RNA negativity at day 3, 5, and 14 in each group, median change of quantitative RT-PCR results, symptomatic improvement, clinical outcomes (duration of hospitalization, ICU stay, intubation, and death), and incidence and severity of adverse events. The severity grading of adverse events was assessed according to the Common Terminology Criteria for Adverse Events version 5.0.

### 2.4. Statistical analysis

The primary analysis compared the proportion of participants who were SARS-CoV-2 negative at day 7 between the two groups using Fisher’s exact test. A key secondary analysis compared the median change in quantitative RT-PCR results from baseline to day 7 between two groups using Mann–Whitney U test. Additional secondary analyses compared the proportion of participants who were SARS-CoV-2 negative at day 3, 5, and 14, and the median change of viral load at day 14 using the same approach at day 7. Statistical analyses were conducted with Prism 9.0 software (GraphPad).

### 2.5. Sample size determination

We anticipated that no participants in the placebo group would be SARS-CoV-2 negative at day 7, and 50% of participants in the peginterferon group would be SARS-CoV-2 negative at day 7. If the proportion of participants who achieve negativity for SARS-CoV-2 is higher than 30% at day 7 in the peginterferon lambda group, the treatment was to be considered for further study. Assuming 50% of participants in peginterferon lambda group are SARS-CoV-2 negative, we estimated that there would be a 94% chance of observing at least 3 out of 10 participants would be SARS-CoV-2 negative at day 7.

## 3. Results

**Figure 1:**
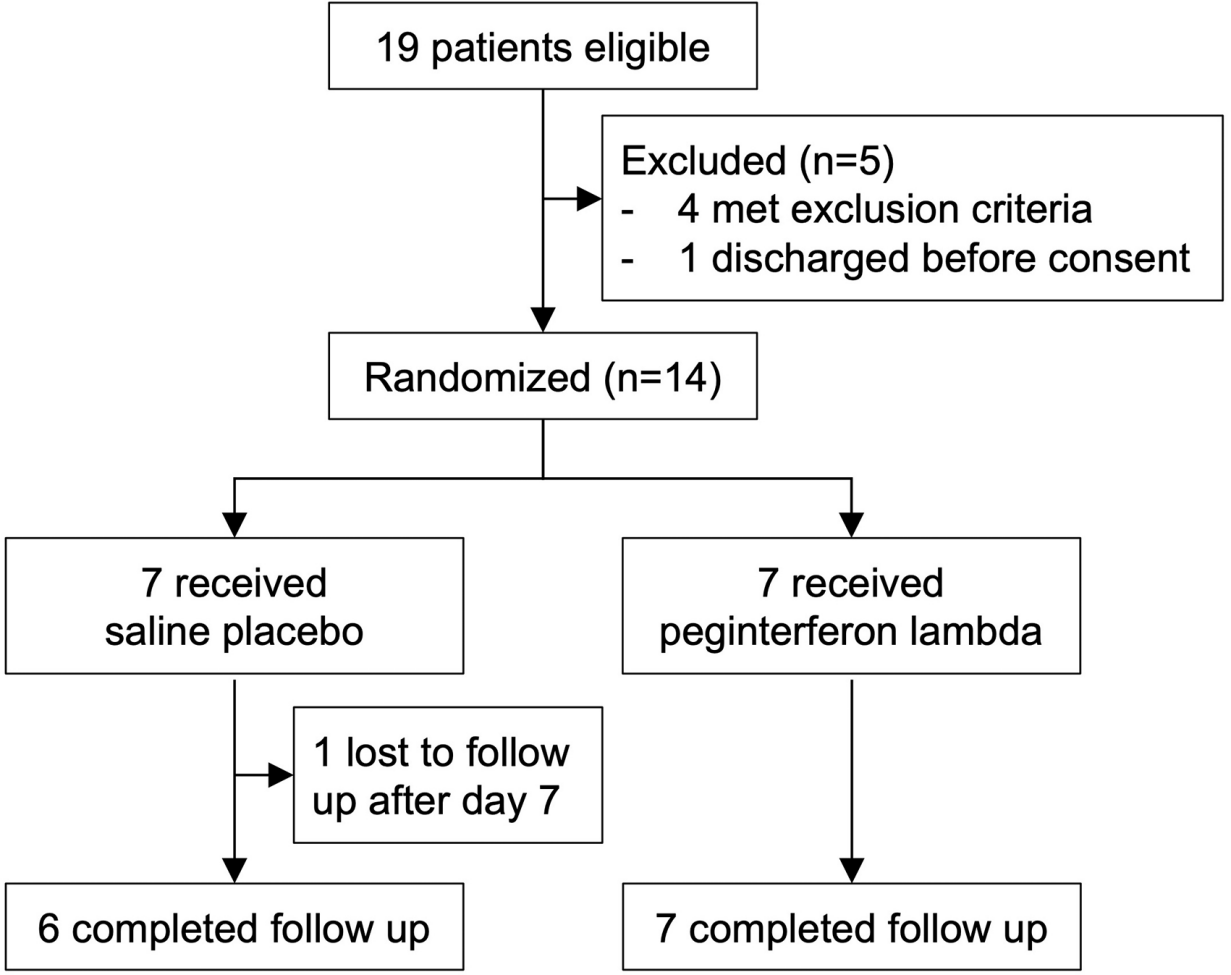
CONSORT diagram.

We enrolled 14 participants admitted to the Massachusetts General Hospital and with COVID-19 between July 14, 2020 and July 16, 2021. The median age was 54.0 (IQR 45.50 to 58.50), 11 participants (78.6%) were male, and 9 (64.3%) were Hispanic (Table 1; Supplementary Table 1). All 14 randomly assigned participants were initially injected with a placebo or peginterferon lambda within a median of 43.1 h (IQR 35.8 to 48.4) after testing SARS-CoV-2 positive. Thirteen participants (92.9%) completed 14 days of follow up with 1 participant in the placebo group lost to follow up after day 7 (Figure 1). The median baseline SARS-CoV-2 viral load was 1.38 log copies per ml (IQR 0.00 to 4.23), with 5 participants (71.4%) in the placebo group and 2 participants (28.6%) in the peginterferon lambda group having undetectable viral load on the day of randomization although they were tested SARS-CoV-2 RNA positive within 48 h of randomization. The median sum of symptom scores was 2.00 (IQR 0.00 to 4.00) in the placebo group and 5.50 (IQR 2.75 to 7.50) in the peginterferon lambda group. Six participants (85.7%) in the peginterferon lambda group and 1 participant (14.3%) in the placebo group were treated with remdesivir and corticosteroids (Table 1).

**Table 1:** Baseline characteristics.

	Placebo (*n* = 7)	Treatment (*n* = 7)	Overall (*n* = 14)
Age	59.00 (49.0–60.5)	54.00 (45.5–58.5)	54.00 (45.5–58.5)
Male	6 (85.7)	5 (71.4)	11 (78.6)
Race, Ethnicity			
White, Hispanic	4 (57.1)	3 (42.9)	7 (50.0)
Unknown, Hispanic	2 (28.6)	0 (0.0)	2 (14.3)
White, Non-Hispanic	1 (14.3)	4 (57.1)	5 (35.7)
BMI, kg/m^2^	27.06 (26.69–31.06)	32.49 (30.60–37.04)	30.88 (26.92–33.11)
Comorbid conditions			
Chronic cardiac disease	3 (42.9)	0 (0.0)	3 (21.4)
Hypertension	5 (71.4)	3 (42.9)	8 (57.1)
Chronic pulmonary disease	1 (14.3)	0 (0.0)	1 (7.1)
Asthma	0 (0.0)	3 (42.9)	3 (21.4)
Obesity	1 (14.3)	5 (71.4)	6 (42.9)
Mild liver disease	1 (14.3)	0 (0.0)	1 (7.1)
Malignant neoplasm	0 (0.0)	1 (14.3)	1 (7.1)
Rheumatological disorder	1 (14.3)	3 (42.9)	4 (28.6)
Diabetes Mellitus	3 (42.9)	0 (0.0)	3 (21.4)
Laboratory values			
AST, U/L	29.00 (22.50–34.00)	37.00 (29.00–57.50)	30.00 (26.00–52.00)
ALT, U/L	25.00 (13.00–50.50)	39.00 (33.50–50.00)	36.50 (25.75–52.75)
Total Bilirubin, U/L	0.40 (0.30–0.45)	0.60 (0.35–0.60)	0.40 (0.30–0.60)
Creatinine, mg/dl	0.87 (0.73–1.00)	0.81 (0.70–0.81)	0.81 (0.70–0.95)
Hemoglubin, g/dl	13.60 (12.45–14.05)	14.40 (13.80–15.10)	13.95 (12.70–14.73)
White blood cells, 10^9^/L	6.29 (5.42–9.63)	6.75 (5.62–8.09)	6.52 (5.34–9.24)
Platelets, 10^9^/L	254.00 (211.00–276.50)	193.00 (169.00–280.50)	247.00 (173.75–286.25)
Lymphocytes, %	12.95 (3.69–24.65)	23.90 (5.66–29.00)	17.60 (2.15–27.30)
Neutrophils, %	64.00 (19.00–66.42)	56.10 (26.78–65.45)	62.90 (6.45–67.10)
SARS-CoV-2 viral load, log10 copies/ml	0.00 (0.00–1.38)	3.60 (1.56–4.58)	1.38 (0.00–4.23)
Negative	5 (71.4)	2 (28.6)	7 (50.0)
Symptom score	2.00 (0.00–4.00)	5.50 (2.75–7.50)	3.00 (2.00–6.00)
Remdesivir	1 (14.3)	6 (85.7)*	7 (50.0)
Duration, days	4	4.5 (3.25–5.75)	4.00 (3.50–5.50)
Corticosteroids	1 (14.3)	6 (85.7)*	7 (50.0)
Duration, days	6	4 (3.00–6.75)	4.00 (3.00–6.00)
Interferon lambda, pg/ml	124.80 (75.75–302.40)	142.20 (96.14–226.10)	131.70 (92.79–193.00)
Interferon alpha, pg/ml	27.14 (14.39–47.66)	53.44 (15.77–92.66)	30.19 (16.14–79.88)
IP-10, pg/ml	256.9 (168.3–433.2)	1,085 (538.6–2,534)*	538.6 (210.8–1,343)
MIG, pg/ml	623.7 (496.8–1,393)	1,836 (1,355–1,995)	1,355 (536.2–1,938)

Data are *n* (%), median (IQR).

* *p* < 0.05 compared with placebo.

### 3.1. Viral kinetics

**Figure 2:**
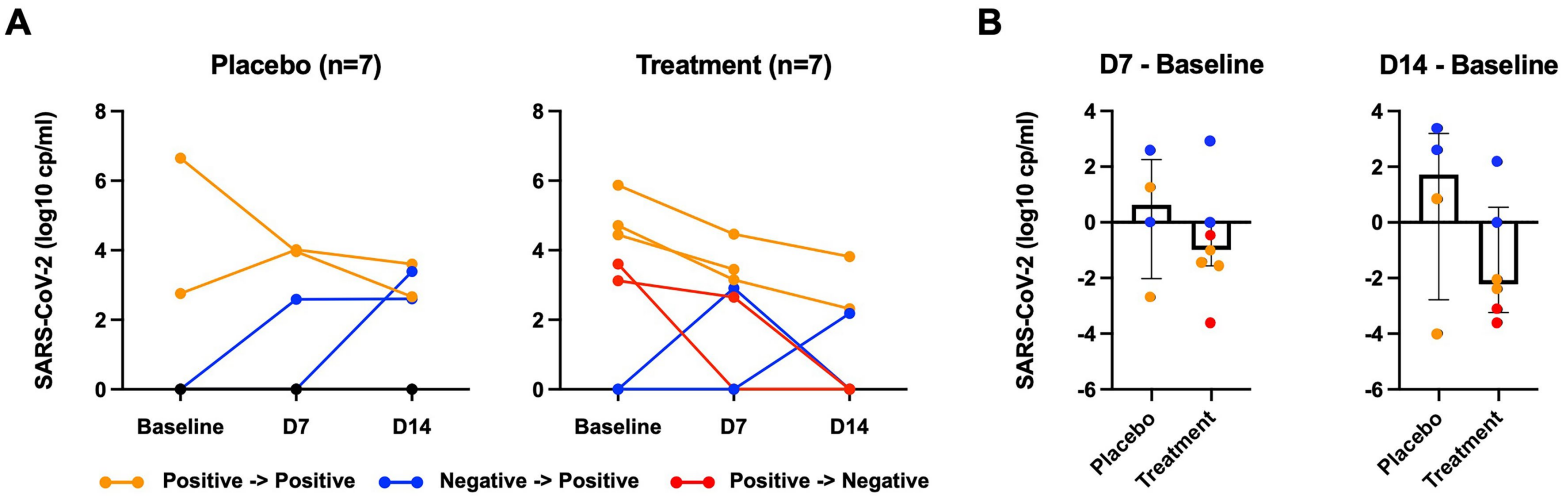
SARS-CoV-2 viral load. **(A)** SARS-CoV-2 viral load of nasopharyngeal swab measured by RT-PCR at baseline, 7 days after baseline (D7), 14 day after baseline (D14). **(B)** SARS-CoV-2 viral load at D7 or D14 were subtracted from those at baseline. Orange color indicates participants who remain SARS-CoV-2 positive; blue color indicates participants who went from negative to positive; red color indicates participants who became SARS-CoV-2 negative.

Among 5 participants who were SARS-CoV-2 positive at baseline in the peginterferon lambda group, 2 participants (40%) became negative at day 14 including 1 participant who achieved negativity for SARS-CoV-2 at day 7, while none (0%) of 2 participants who were SARS-CoV-2 positive at baseline in the placebo group achieved negativity for SARS-CoV-2 by day 14 (*p* > 0.05). Two of 5 participants who were SARS-CoV-2 negative at baseline in the placebo group became positive at day 14 including 1 participant who had already tested positive at day 7. Among 2 participants who were SARS-CoV-2 negative at baseline in the peginterferon lambda group, one participant became positive at day 14 and the other participant became positive at day 7 but turned negative at day 14 (Figure 2A). When 3 participants who remained SARS-CoV-2 negative throughout the study in the placebo group were excluded, the median change of viral load (log copies per ml) at day 7 was +0.63 (IQR −2.02 to 2.56) in the placebo group and − 0.99 (IQR −1.56 to 0.00) in the peginterferon lambda group (*p* = 0.20), and +1.72 (IQR −2.78 to 3.19) in the placebo group and −2.22 (IQR −3.24 to 0.55) in the peginterferon lambda group at day 14 (*p* = 0.24; Figure 2B).

### 3.2. Cytokine and chemokine levels

**Figure 3:**
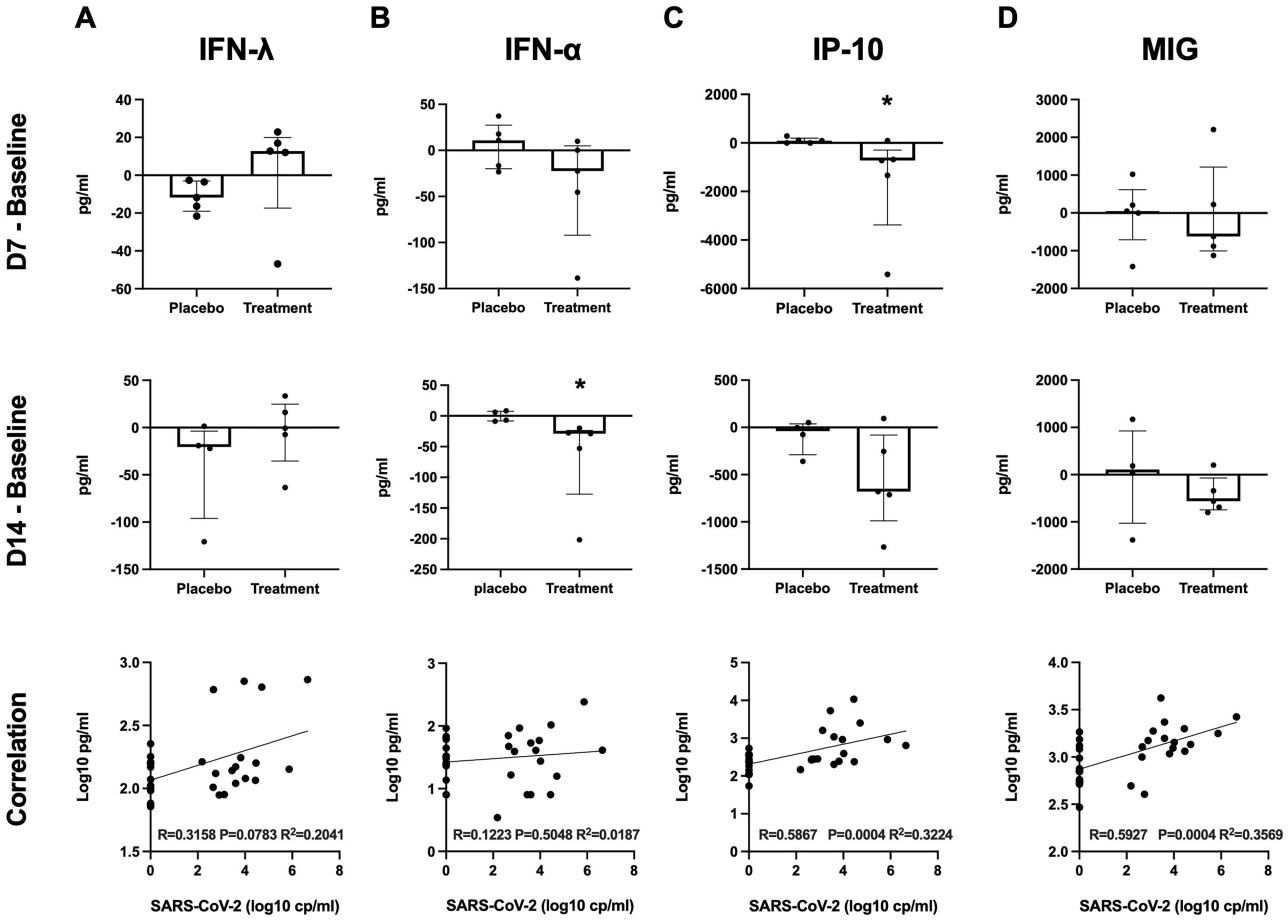
Plasma cytokines. Plasma cytokine levels of interferon lambda **(A)**, interferon alpha **(B)**, IP-10 **(C)**, and MIG **(D)** at D7 or D14 were subtracted from those at baseline. Significance was determined using Mann–Whitney U test. *p < 0.05 Spearman rank test was used for correlations. Solid lines represent regression lines.

The levels of plasma interferon lambda (pg/ml) at baseline were not significantly different between the placebo group and peginterferon lambda group (median 124.8 and 142.2, respectively, *p* = 0.36; Table 1). The median change of the plasma interferon lambda levels at day 7 from baseline was −11.77 (IQR −19.00 to −3.08) in the placebo group and +12.75 (IQR −17.39 to 19.92) in the peginterferon lambda group (*p* = 0.07), and −20.55 (IQR −96.11 to −3.7) in the placebo group and −0.71 (IQR −35.35 to 24.87) in the peginterferon lambda group at day 14 from baseline (*p* = 0.14; Figure 3A). The levels of plasma interferon alpha (pg/ml) at baseline were not significantly different between the placebo group and peginterferon lambda group (median 27.14 and 53.44, respectively, *p* = 0.47; Table 1). The median change of the plasma interferon alpha levels at day 7 from baseline was +10.99 (IQR −19.90 to 27.48) in the placebo group and −22.44 (IQR −92.02 to 4.96) in the peginterferon lambda group (*p* = 0.14), and −0.43 (IQR −8.13 to 7.72) in the placebo group and −28.87 (IQR −127.3 to −23.71) in the peginterferon lambda group at day 14 from baseline (*p* = 0.01; Figure 3B). The baseline plasma IP-10 level (pg/ml) of the peginterferon lambda group was higher than that of the placebo group (median 1,085 and 256.9, respectively, *p* = 0.03; Table 1). The median change of the plasma IP-10 levels at day 7 from baseline was +87.51 (IQR −5.9 to 198.2) in the placebo group and −723.2 (IQR −3,377 to −298.4) in the peginterferon lambda group (*p* = 0.03), and −38.92 (IQR −288.4 to 38.04) in the placebo group and −679.9 (IQR −988.8 to −80.22) in the peginterferon lambda group at day 14 from baseline (*p* = 0.14; Figure 3C). The baseline plasma MIG level (pg/ml) of the peginterferon lambda group appeared to be higher than that of the placebo group (median 1836 and 623.7, respectively, *p* = 0.12; Table 1). The median change of plasma MIG levels at day 7 from baseline was +47.54 (IQR −712.0 to 614.0) in the placebo group and −624.0 (IQR −1,004 to 1,214) in the peginterferon lambda group (*p* = 0.50), and +108.1 (IQR −1,028 to 926.3) in the placebo group and −563.2 (IQR −746.4 to −70.11) in the peginterferon lambda group at day 14 from baseline (*p* = 0.28; Figure 3D). The levels of IP-10 and MIG, but not interferon lambda and interferon alpha, were correlated with SARS-CoV-2 viral load (Figures 3A–D, bottom).

### 3.3. Symptoms and clinical outcomes

**Figure 4:**
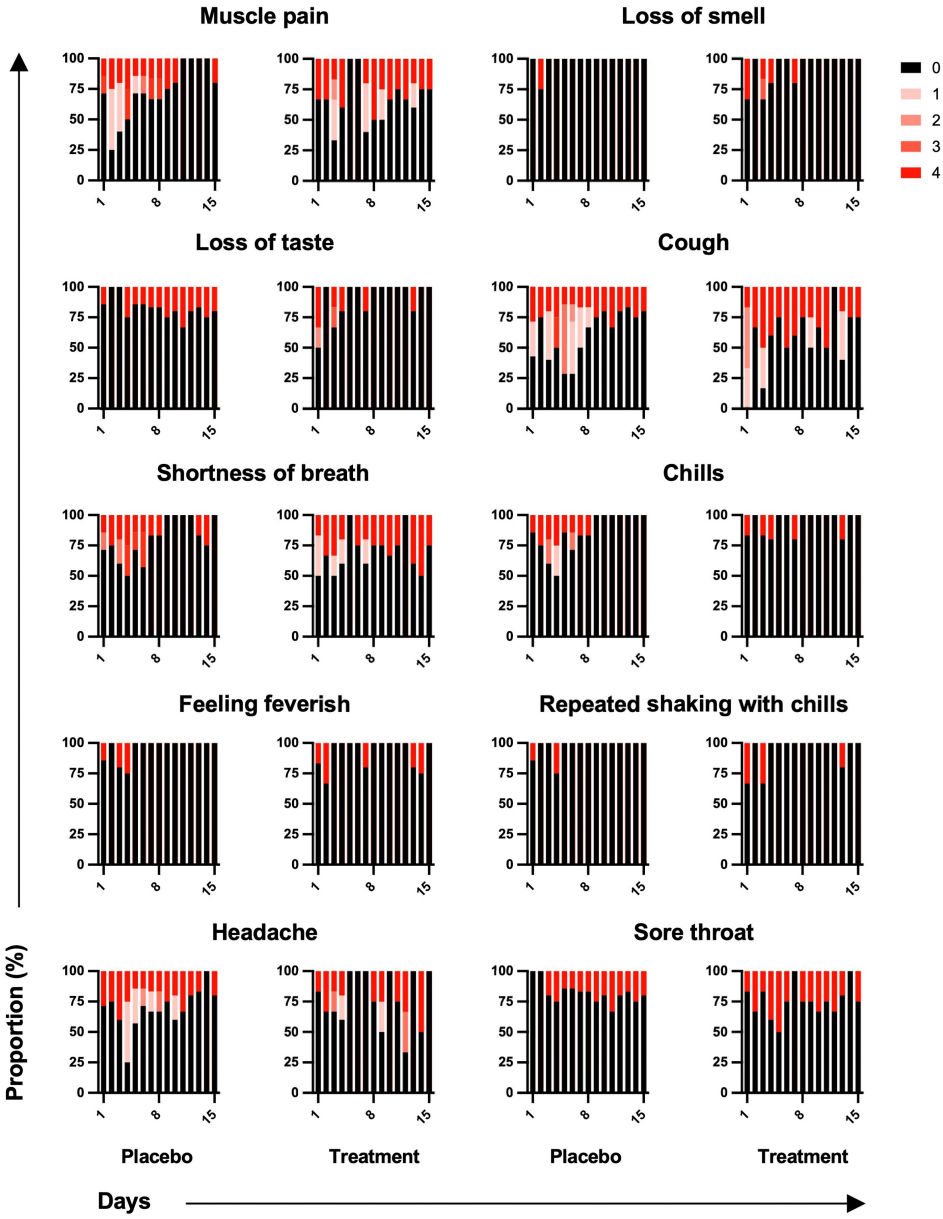
Symptom changes. Symptoms related to COVID-19 were reported as not at all, a little bit, somewhat, quite a bit, or very much and scored on a scale of 0 to 4, respectively.

**Figure 5:**
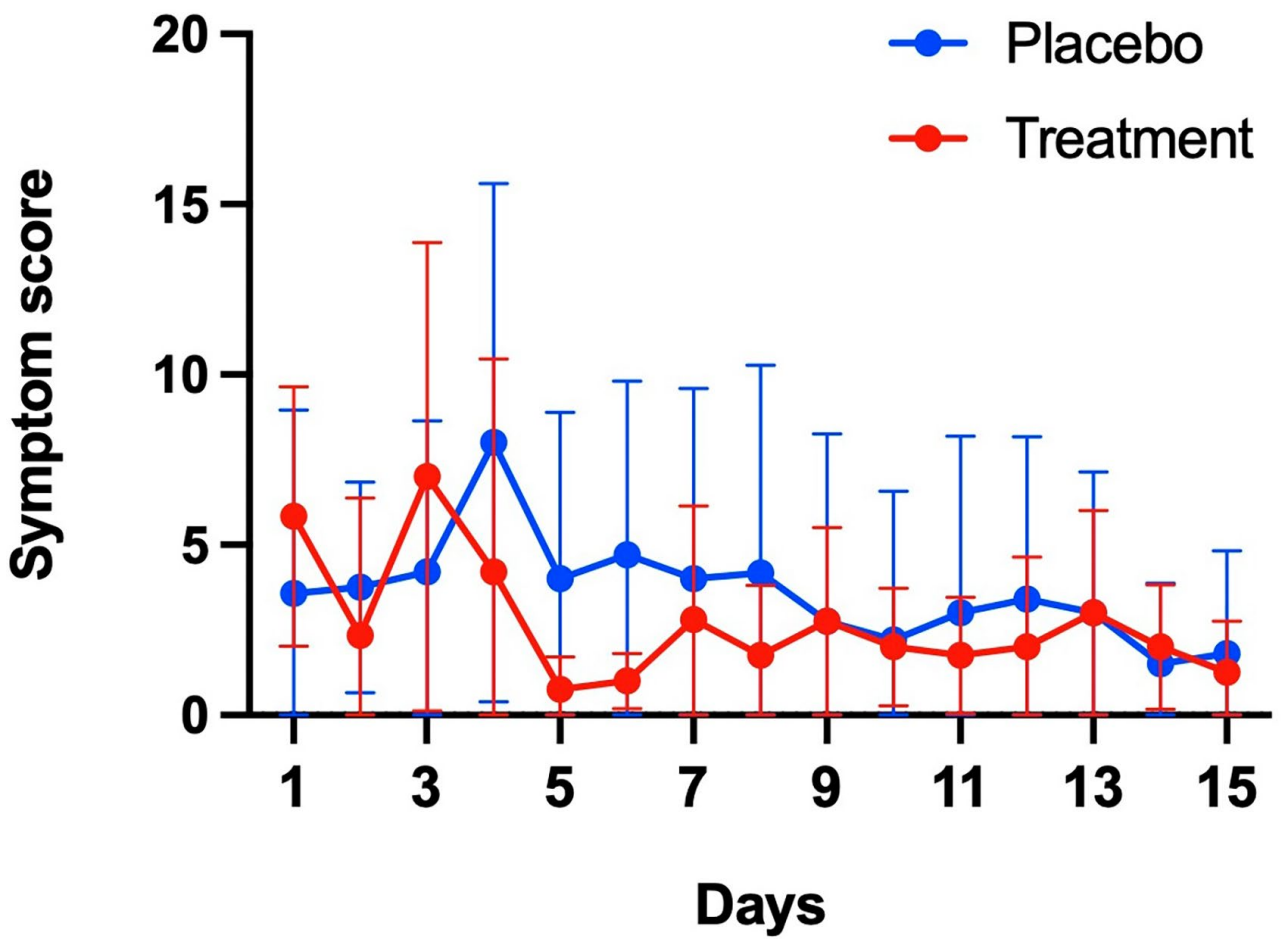
Symptom changes. Sum of 10 symptom scores were compared between placebo and treatment groups.

Among 10 symptoms related to COVID-19, muscle pain, cough, shortness of breath, and headache were common symptoms in both groups (Figure 4). Although symptoms in both groups appeared to subside over time, the sum of symptom scores did not differ significantly between the two groups (Figure 5). The duration of hospitalization was similar between two groups: a median of 4 days (IQR 3 to 8) in the placebo group and 5 days (IQR 4 to 8) in the peginterferon lambda group. There were no ICU events or deaths other than one participant in the peginterferon lambda group receiving ICU care for pneumonia that required intubation, which was considered unrelated to the study drug.

### 3.4. Adverse events

Two serious adverse events were reported in the peginterferon lambda group, where pneumonia requiring intubation and transaminitis were considered unrelated or possibly related to the study drug, respectively. There were ICU delirium (unrelated), stomach pain (possibly related), and transaminitis (probably related) in the peginterferon lambda group, while 1 participant in the placebo group reported shortness of breath and chest pain unlikely related to the study drug. Study drug was discontinued for the participants who experienced transaminitis and pneumonia, and all participants recovered from their adverse events (Table 2).

**Table 2:** Summary of adverse events.

Relationship to study drug	Adverse event term	Severity	Study drug	Group
Unrelated	ICU Delirium	Grade 2	Not changed	Treatment
Unrelated	*Worsening pneumonia requiring intubation	Grade 3	Discontinued	Treatment
Possibly related	Stomach pain	Grade 2	Not changed	Treatment
Possibly related	*Transaminitis	Grade 4	Discontinued	Treatment
Unlikely related	Shortness of breath, chest pain	Grade 2	Not changed	Placebo
Probably related	Transaminitis	Grade 3	Discontinued	Treatment

* Serious adverse event.

## 4. Discussion

In patients initially admitted to the hospital with COVID-19, treatment with subcutaneous injection of peginterferon lambda every 7 days may accelerate viral load decline and decrease plasma levels of IP-10 and MIG. Peginterferon lambda was well tolerated, although there were a few study drug-related adverse effects such as transaminitis.

Recently, 2 clinical trials evaluated the efficacy and safety of peginterferon lambda reducing SARS-CoV-2 viral load in outpatients with COVID-19 (6, 7). Feld et al. demonstrated that peginterferon lambda accelerates viral load decline in outpatients with COVID-19, particularly in those with high baseline viral load above 6 log copies per ml (6), while Jagannathan et al. did not find significant efficacy of peginterferon lambda in shortening the duration of SARS-CoV-2 viral shedding regardless of baseline viral load (7). In our study, peginterferon lambda appeared to accelerate SARS-CoV-2 viral load decline compared to the placebo group even though the baseline viral loads were under 6 log copies per ml in both groups. Six of seven participants in the peginterferon lambda group were treated with remdesivir for a median of 4.5 days before and after enrollment, and the other participant who became SARS-CoV-2 positive at day 14 was not treated with remdesivir, while only 1 participant in the placebo group was treated with remdesivir. There are few trials that have reported the effect of remdesivir on viral kinetics in COVID-19 (8–10), although remdesivir was found to lead to modest but significant improvement in symptoms of COVID-19 (11). Given that several combination therapies using remdesivir and immunomodulating drugs are being tested (12), further studies should examine whether combination therapy using remdesivir and peginterferon lambda might have stronger efficacy reducing SARS-CoV-2 viral load than monotherapy.

The plasma level of interferon lambda appeared to be increased in the peginterferon lambda group as expected, but the degree of the increase was relatively small, except for one subject in whom the plasma level of interferon lambda were particularly high at baseline and declined steeply (Supplementary Figure 1). The plasma levels of interferon lambda and interferon alpha were not associated with SARS-CoV-2 viral load, suggesting the increase of plasma interferon lambda is due to the introduced peginterferon lambda. A pharmacokinetic study of peginterferon lambda following a 180 mcg subcutaneous administration demonstrated that the mean Cmax was 1.00 ng/ml (standard deviation 109) and the median Tmax was 12 h (IQR 8 to 120 h) in healthy subjects (13). Thus, the degree of increase in plasma interferon lambda should be small at day 7 and day 14. In addition, a 180 mcg dose of peginterferon lambda might not provide sufficient therapeutic efficacy in reducing viral load. In an *in vitro* model of SARS-CoV-2 infection, human epithelial cells treated with more than 37 ng/ml of peginterferon lambda had a considerable viral load decline. In a mouse model of SARS-CoV-2, 2 mcg subcutaneous administration of peginterferon lambda resulted in considerable decreases of SARS-CoV-2 viral loads (14). However, the 2 mcg dose in mice is a 3-fold larger dose than 180 mcg dose in humans based on human equivalent dose (15). Even though higher or more frequent dosing might be advantageous, a dose greater than 180 mcg in humans should be limited as it increases drug toxicity, including transaminitis (16). Therefore, a possible role of peginterferon lambda in reducing viral load may be to potentiate antiviral effects when combined with remdesivir or another antiviral agent.

We found that the levels of plasma IP-10 and MIG appeared to be reduced in the peginterferon lambda group. IP-10 and MIG are induced in viral infection and stimulate immune cells to fight against viral infection; on the other hand, they may also potentially induce hyperinflammation, making IP-10 and MIG markers of COVID-19 disease progression (17, 18). The reduced levels of IP-10 and MIG suggests that the patients in the peginterferon lambda group would experience favorable outcomes. The correlation between the SARS-CoV-2 viral loads and the plasma levels of IP-10 and MIG supports the finding of viral load decline in the peginterferon lambda group. However, it should also be noted that most of the patients in the peginterferon lambda group received corticosteroids treatment, which also can reduce the level of IP-10 in severely ill COVID-19 patients (19).

In general, it has been reported that interferon therapy can be associated with hepatotoxicity (20); still, few studies reported that peginterferon lambda therapy is associated with transaminitis (4, 5). The previous two clinical trials of peginterferon lambda in outpatients with COVID-19 also reported that transaminase elevation was frequent in the peginterferon lambda group, but there were no clinical consequences and lab abnormalities were not sustained (6, 7), which was also observed in this study.

This study did have a few limitations. First, we had limited number of subjects. This was a pilot study designed to include 10 subjects in each arm to evaluate the efficacy of peginterferon lambda to reduce SARS-CoV-2 viral load in hospitalized patients with COVID-19. We enrolled seven participants instead of 10 in each arm because of premature termination due to constraints imposed on our enrollment once vaccines became available to exclude vaccinated persons, which substantially hampered enrollment. Second, because there were participants who were SARS-CoV-2 negative at baseline and possibly false positive for SARS-CoV-2 at screening, it was not possible to include all participants together in the statistical analysis. If the date of symptom onset was considered at the time of enrollment, we could uniformly include the participants positive for SARS-CoV-2 at baseline. Third, since most of the patients in the peginterferon lambda group received remdesivir treatment, it is challenging to determine the efficacy of peginterferon lambda alone. When we designed this study, remdesivir receipt was not designated an exclusion criterion, since it was widely being used and it was expected that there would be balanced use between arms. The discrepancy in the number of subjects treated with remdesivir between two groups suggests that there might be imbalances in disease severity at baseline, which is supported by the observation that the symptom scores and plasma levels of IP-10 and MIG (COVID-19 disease progression markers as mentioned above) were lower in the placebo group. Fourth, for enrollment, we reviewed patients within 24 h of hospitalization and within 48 h of diagnosis with COVID-19. These may have included patients who were hospitalized for other reasons but also had COVID-19. Finally, this study was conducted in a single-blind design, but it is unlikely to affect objective outcome evaluations such as SARS-CoV-2 viral load and plasma cytokine levels.

In conclusion, in this small single-center pilot trial peginterferon lambda treatment appears to accelerate SARS-CoV-2 viral load decline and improve the plasma disease progression markers in hospitalized patients with COVID-19. These results should be factored into decisions to pursue further phase 2 and 3 trials for the use of peginterferon lambda in patients with COVID-19.

## Data availability statement

The original contributions presented in the study are included in the article/Supplementary material, further inquiries can be directed to the corresponding author.

## Ethics statement

The studies involving human participants were reviewed and approved by Massachusetts General Hospital. The patients/participants provided their written informed consent to participate in this study.

## Author contributions

MHK, NJ, AK, and RC contributed to study conception and design. MHK, JE, JG, and EN contributed to data acquisition. MHK and RC contributed to the drafting of the manuscript. NJ, MX, DH, and AK contributed to critical revision of the manuscript. All authors contributed to the article and approved the submitted version.

## Funding

This work was supported by Massachusetts General Hospital Research Scholars Program; and the Jacob Soumerai Research Sundry Fund.

## Conflict of interest

The authors declare that the research was conducted in the absence of any commercial or financial relationships that could be construed as a potential conflict of interest.

## Publisher’s note

All claims expressed in this article are solely those of the authors and do not necessarily represent those of their affiliated organizations, or those of the publisher, the editors and the reviewers. Any product that may be evaluated in this article, or claim that may be made by its manufacturer, is not guaranteed or endorsed by the publisher.
