# Supplementary material for: Peginterferon lambda for the treatment of hospitalized patients with mild COVID-19: A pilot phase 2 randomized placebo-controlled trial
Source: Front Med (Lausanne). 2023 Feb 24;10:1095828. doi: 10.3389/fmed.2023.1095828 (PMC10002416; doi:10.3389/fmed.2023.1095828)
Supplement: Supplementary file 1 [file Data_Sheet_1.PDF]

MASSACHUSETTS GENERAL HOSPITAL
LIVER CENTER

CLINICAL STUDY PROTOCOL

**Study Title:**	A Randomized Controlled Trial to Evaluate the Safety and Efficacy of Pegylated Interferon Lambda vs. Placebo in Subjects Infected with COVID-19
**Sponsor:**	Raymond Chung, MD<br>Massachusetts General Hospital<br>Boston, MA 02114
**IND Number:**	149760
**ClinicalTrials.gov Identifier**	NCT04343976
**Protocol ID:**	2020P001083
**Sponsor/Emergency Contact**	Raymond Chung, MD<br>Gastrointestinal Unit<br>Massachusetts General Hospital<br>55 Fruit Street; Boston MA 02114<br>(P): 617-724-7562<br>(E): Chung.Raymond@mgh.harvard.edu
**Co-PI**	Arthur Kim, MD<br>Infectious Diseases Unit<br>Massachusetts General Hospital<br>akim1@mgh.harvard.edu
**Co-Investigator**	Nikolaus Jilg, MD, PhD<br>Infectious Diseases Unit<br>Massachusetts General Hospital<br>njilg@partners.org
**Co-Investigator**	Ashwini Arvind, MBBS<br>Gastrointestinal Unit<br>Massachusetts General Hospital<br>darvind@mgh.harvard.edu
**Protocol Version/Date:**	Version 1.2: 26 April 2020

Contents

Abbreviations **4**

I. Introduction **5**

II. Objectives **6**

Primary Objectives 6

Secondary Objectives 6

III. Study Design **6**

Visit schedule 6

Administration of Treatment 6

Stopping Rules and Treatment Discontinuation 7

Toxicity Based Stopping Criteria 7

IV. Subject Population **7**

Inclusion Criteria 7

Exclusion Criteria 8

V. Investigational Product **8**

Randomization, Blinding and Treatment Codes 8

Description and Handling of Lambda 9

Prior and Concomitant Medications 9

Accountability for Lambda 10

Investigational Product Return or Disposal 10

VI. Study Procedures **10**

Subject Enrollment and Treatment Assignment 10

Pretreatment Assessments 10

Baseline Assessments ...... **Error! Bookmark not defined.**

Post-treatment Assessments 12

Unscheduled Visits 12

Early Termination Visit 13

VII. Adverse Events and Toxicity Management **13**

Definition of Adverse Events, Adverse Reactions and Serious Adverse Events 13

Adverse Event 13

Serious Adverse Event 13

Clinical Laboratory Abnormalities and Other Abnormal Assessments as Adverse Events or Serious Adverse Events 14

Assessment of Adverse Events and Serious Adverse Events 14
Assessment of Causality for Study Drugs and Procedures 14
Assessment of Severity 15
Reporting of Adverse Events 15
VIII. Statistical Considerations 15
Data Analysis 15
Sample size and power calculation: 16
IX. References 17
Appendices 18
Appendix 1: Investigator Signature Page 19
Appendix 2: Study Procedures Table 20
Appendix 3: GIS Grading Scale for Severity of Adverse Events and Laboratory Abnormalities 21
Appendix 4: Pregnancy Precautions and Definitions 22
Appendix 5: Symptom Questionnaire 23

Abbreviations

AE	Adverse Event
CBC	Complete Blood Count
LFT	Liver Function Test
CRF	Case Report Form
CRP	C-Reactive Protein
eCRF	Electronic Case Report Form
ET	Early Termination
IMP	Investigational Medicinal Product
IRB	Institutional Review Board
PI	Principal Investigator
PICC	Peripherally inserted central catheter line
RCT	Randomized Controlled Trial
RIC	Respiratory Illness Clinic
SAE	Serious Adverse Event
SARS	Severe Acute Respiratory Syndrome
SC	Subcutaneous
WOCBP	Women of Childbearing Potential
BMP	Basic Metabolic Panel
AST	Aspartate Aminotransferase
ALT	Alanine Transaminase
AP	Alkaline Phosphatase
TBili	Total Bilirubin
TCRC	Translational and Clinical Research Center
DBili	Direct Bilirubin

I. Introduction

A novel strain of coronavirus (COVID-19) was recently detected in Wuhan, China and reported to the World Health Organization Country Office in China on December 31st 2019. Although respiratory symptoms are absent or mild in the majority of patients, COVID-19 infection is associated with a 4% mortality rate due to the development of acute respiratory distress syndrome, mainly among elderly individuals and those with comorbid conditions. Despite global efforts to contain viral spread, the incidence of COVID-19 continues to rise exponentially. The United States has entered an indefinite period of lockdown, with the current crisis likely to incur an immense socioeconomic burden. At present, there are no vaccines available for disease prevention. Therefore, identifying effective treatment strategies to curb human-to-human transmission, while reducing disease severity and shortening hospital and ICU admissions, is an area of urgent and unmet clinical need.

Peginterferon lambda-1a (Lambda) is a conjugate of recombinant human interleukin 29 (rIL-29) and a linear polyethylene glycol (PEG) chain, and has been identified as a therapeutic candidate. Lambda has been extensively trialled as a treatment for chronic hepatitis B, C and D, and its safety and tolerability have been demonstrated for up to 48 weeks of treatment, with over 3000 patients dosed with an excellent safety record. Lambda targets type III IFN receptors, whose expression is primarily restricted to the lungs, intestine, and liver. This is likely to account for the fewer off-target effects associated with use of Lambda versus other trialled interferons.

Murine studies also indicate robust antiviral effects of IFN lambdas against SARS-CoV, influenza, rotavirus, norovirus and reovirus[1]. Furthermore, SARS-CoV replicated to high titers and induced signs of disease in mice lacking both type I and type III IFN receptors. These effects were not observed in single-knockout mice expressing either type I or type III IFN receptors, indicating protective roles for both type I and type III IFNs against SARS-CoV[2]. In addition, treatment of influenza A-infected mice with IFN lambda significantly increased their survival and reduced morbidity. Treatment with IFN alpha demonstrated comparable antiviral activity, however it exerted proinflammatory effects on immune cells, with an associated increase in mortality. ***Hence, IFN lambda derivatives may represent a superior treatment option compared to IFN alpha.***

The potential benefit of IFN-based therapy in the management of coronavirus infection was evaluated in a preliminary study of patients admitted to hospital with probable SARS-CoV in 2003[3]. Administration of IFN alfacon-1, a synthetic recombinant type I IFN, plus corticosteroids was associated with more rapid resolution of radiographic lung abnormalities and improved oxygen saturation levels compared to treatment with corticosteroids alone.

On the basis of these preliminary findings, it is highly plausible that treatment with Lambda will promote COVID-19 clearance and improve clinical outcomes. However, to date, evidence in humans is lacking. We propose to evaluate the safety, efficacy and tolerability of Lambda treatment versus standard of care in patients admitted to hospital with confirmed COVID-19 infection. We will conduct a randomized controlled trial (RCT) of hospitalized subjects diagnosed with COVID-19 infection by PCR analysis of nasopharyngeal secretions. Subjects will be randomized 1:1 to receive Lambda 180 mcg SC or saline injection The primary endpoint is rate of PCR negativity on post-treatment Day 7.

II. Objectives

Primary Objectives

The primary objective of this study is as follows:

- To assess the efficacy of Lambda (180 mcg SC injections) vs. saline placebo injection in inducing PCR negativity at day 7 among subjects admitted to the hospital with COVID-19 infection

Secondary Objectives

The secondary objectives of this study are as follows:

- To assess the efficacy of Lambda (180 mcg SC injections) vs. saline placebo injection in inducing PCR negativity at days 3, 5, 7 and 14 among hospitalized subjects with symptomatic COVID-19 infection
- To assess the efficacy of Lambda (180 mcg SC injections) vs. saline placebo injection in inducing symptomatic improvement in subjects with COVID-19 infection, assessed by daily symptom questionnaire
- To assess the efficacy of Lambda (180 mcg SC injections) vs. saline placebo injection in improving clinical outcomes (death, ICU stay, intubation) in subjects hospitalized with COVID-19 infection
- To evaluate the safety and tolerability of Lambda (180 mcg SC injections) vs. saline placebo injection

III. Study Design

This is a prospective randomized trial to assess the antiviral efficacy of Lambda (180 mcg SC injection) vs. standard of care in up to 20 subjects with COVID-19 infection.

Visit schedule

After referral by the care team and informed consent, and once the subject is confirmed as COVID-19 positive on clinical testing, patients will have initial clinical lab testing performed during baseline (visit 1). After confirmation of inclusion criteria, patients will be randomized 1:1 to receive the first dose of Lambda or saline placebo at the baseline visit (visit 1). Nasal swabs for PCR analysis will be collected on Days 3, 5, 7 and 14. On day 7, subjects in the treatment arm will receive a second Lambda injection and subjects in the control arm will receive a second saline placebo injection. On Day 14, subjects will undergo laboratory measurements to confirm CBC and LFT values are within safe parameters, and to measure levels of pro-inflammatory cytokines (e.g. IFN- γ, IL-6, IP-10, MCP-1, TNF-α). Measurements will be performed using blood collected for clinical purposes wherever possible. Vital signs will be monitored thrice daily. Subjects will complete a daily symptom questionnaire from screening through Day 14 (Appendix 5).

Administration of Treatment

An initial 30 inpatients will be enrolled (consented), with a goal of 20 subjects randomized 1:1 to receive either Lambda 180 mcg SC injections or saline placebo injections. Administration of first dose Lambda/placebo in relation to initial COVID-19 symptom onset will be documented in all subjects.

Stopping Rules and Treatment Discontinuation

If a subject discontinues study dosing (for example, as a result of an adverse event [AE]), every attempt should be made to keep the subject in the study, and continue to perform the required study-related follow-up procedures (Appendix 2). If this is not possible or acceptable to the subject or Investigator, the subject may be withdrawn from the study.

There is no option for Lambda dose reduction due to toxicity. If Lambda is withheld due to toxicity, the subject must discontinue treatment and be approached for an Early Termination (ET) visit as soon as possible. For subjects discharged from the hospital, this visit should be conducted over telephone. An in-person evaluation may be scheduled if this is deemed necessary by the overall study PI.

When medically feasible, the PI must be consulted prior to subject discontinuation.

Study drug must be discontinued in the following instances:

- Unacceptable toxicity (as defined below) or toxicity that, in the judgment of the investigator, compromises the ability to continue study-specific procedures or is considered to not be in the subject's best interest
- Pregnancy in a female subject
- Significant protocol violation that impacts subject safety
- Subject request to discontinue for any reason; it is important to determine whether the withdrawal of consent is primarily due to an AE, lack of efficacy, or other reason
- Subject noncompliance
- Discontinuation of the study at the request of the study sponsor, NIH, regulatory agency or an Institutional Review Board (IRB)

Toxicity Based Stopping Criteria

Subjects who meet any of the following laboratory or adverse event criteria must stop treatment with study drug:

- Any Grade 4 adverse event assessed as related to study drug

IV. Subject Population

Inclusion Criteria

- Admitted to the hospital with Covid-19 infection
- Willing and able to provide informed consent
- Male or female age $\geq$ 18 years at time of consent
- Diagnosed with COVID-19 infection by PCR analysis of respiratory secretions
  - Positive SARS-CoV-2 RT-PCR test must be within 48 hours of randomization
- Female subjects of childbearing potential who engage in heterosexual intercourse must agree to use protocol specified method(s) of contraception as described in Appendix 4
- Subject must be able to comply with the dosing instructions for study drug administration and able to complete the study schedule of assessments

Exclusion Criteria

- Current or prior history of any of the following:
  - Clinically-significant illness or any other major medical disorder that in the opinion of the investigator, may interfere with subject treatment, assessment or compliance with the protocol
  - Treatment with IFN or other immunomodulatory/immunosuppressive agent within 12 months before screening.
  - Respiratory compromise requiring ventilatory support other than nasal cannula (mask, bipap or intubation and mechanical ventilation).
  - History of treatment with any of the following medications within five half-lives or 30 days before administration of the study drug (whichever is longer): anti-IL-6, anti-IL6R antagonists, Janus kinase inhibitors, ustekinumab (anti-IL-12/23), or anti IL-23 agents (guselkumab).
- Pregnant or Nursing Females (a negative serum pregnancy test is required during screening [if historic negative result within 48 hours prior to consent is not available] for WOCBP)
- Life threatening SAE during the screening period
- Subjects having the following laboratory parameters at screening
  - Platelet count <90,000 cells/mm3
  - WBC count <3,000 cells/mm3
  - ANC <1,500 cells/mm3
  - Hb <11 g/dL for women and <12 g/dL for men
  - CrCl < 50 mL/min
  - Bilirubin level ≥ 2.5 mg/dL
  - Alb <3.5 g/dL
  - INR ≥1.5 (except in the setting of concomitant anticoagulant use)
  - D-dimer >1500 ng/mL
  - CRP >200 mg/L
- Females who may wish to become pregnant and/or plan to undergo egg harvesting during the course of the study and up to 30 days of the last dose of study drug. WOCBP must abstain from breastfeeding and be willing to use effective birth control during through the week 4 post treatment follow-up visit.
- Clinically-relevant alcohol or drug abuse within 12 months of screening
- Use of any prohibited concomitant medications as described in Section V
- Known hypersensitivity to IFN
- Current or planned participation in an investigational new drug (IND) trial from 30-days prior to randomization through Day 14 post treatment.

V. Investigational Product

Randomization, Blinding and Treatment Codes

This is a randomized, single-blinded controlled study. Subjects will be randomized 1:1 to receive Lambda 180 mcg SC injections vs. saline placebo injections.

Description and Handling

Lambda Formulation

Lambda injection is a sterile, nonpyrogenic, ready to use solution (0.4 mg/mL) that is clear to opalescent, colorless to pale yellow, and is essentially free of particles. Lambda injection is provided in a 1-mL long Type I glass syringe with a 29-gauge, ½-inch, thin-walled needle. The syringes are prefilled with a solution of Lambda Injection, mannitol, L-histidine, polysorbate 80, hydrochloric acid, and water for injections; they are intended for a single use (180 mcg dose). The study drug will be donated by Eiger Biopharmaceuticals. Swab kits and RT-PCR testing will be supplied by a contract research organization, Viroclinics.

Lambda Packaging and Labeling

Storage of the study drug will be done in the Massachusetts General Hospital Clinical Trials Pharmacy (MGH CTP) (directed by Dr. John Vetrano, PharmD). Study drug will be packaged and labeled per the Massachusetts General Hospital Investigational Pharmacy standard procedure and will adhere to federal guidelines.

Lambda Storage and Handling

Prefilled syringes should be stored in a refrigerator at 2 - 8°C (36 - 46°F) and protected from long-term (>24 hours) exposure to light. Lambda Injections should not be frozen. Until dispensed to the subjects, syringes containing the study drug should be stored in a securely locked area, accessible only to authorized site personnel. To ensure the stability and proper identification, study drugs should not be stored in a container other than the container in which they were supplied.

Saline Placebo

In this single blind study, saline placebo injections will be prepared by the MGH CTP. Under the direction of the lead pharmacist, pharmacy staff will prepare the placebo injections by drawing up approximately 0.45 ml of sterile saline into a 1 ml Monoject Tuberculin Safety Syringe with a 28 gauge, ½ inch, thin walled needle. Placebo injections will be labeled with standard MGH research prescription EPIC label as peginterferon lambda-1a or placebo however the display name and administration instructions will be unblinded for provider to be able to choose the correct treatment.

Prior and Concomitant Medications

Concomitant medications taken within 30 days prior to screening, up to and including 30 days after the last dose of study drug, need to be recorded in the source documents and electronic case report form(s) (eCRFs).

In drug-drug interaction studies in healthy subjects, the activity of select CYP enzymes were evaluated and suggest that following a single 180µg dose, Lambda is a mild inhibitor of CYP1A2, CYP2C9, and CYP3A4 and a moderate inhibitor of CYP2C19 and CYP2D6. Because the effects of these sensitive CYP substrates are mild or moderate, dose adjustments for other concomitant CYP substrates may not be necessary, but should be used with caution.

Accountability for Lambda/Placebo

The investigator or designee (i.e. pharmacist) is responsible for ensuring adequate accountability of all used and unused study drug. This includes acknowledgement of receipt of each shipment of study drug (quantity and condition). Used study drug will be disposed of in the appropriate sharps container. Administration of Lambda or saline placebo injections will be performed by site staff and accountability of used medication will be documented in the source.

Lambda/placebo accountability records will be utilized to:

- Record the date received and quantity of study product
- Record the date, subject number, subject initials, the study product dispensed
- Record the date, quantity of used and unused study product returned, along with the initials of the person recording the information.

Investigational Product Return or Disposal

Due to the nature of the study product (prefilled syringes for injection), used study medication will not be returned to the study team or research pharmacy and should be disposed of in the appropriate sharps container. The MGH Clinical Trials Pharmacy Established Site Plan will be followed in regard to disposal of unused investigational product.

VI. Study Procedures

The study procedures to be conducted for each subject enrolled in the study are presented in tabular form in Appendix 2 and described in the text that follows.

The investigator must document any deviation from protocol procedures and notify the lead PI and IRB.

Subject Enrollment and Treatment Assignment

Study staff, along with treating providers on the Inpatient team, will review current admitted patient charts to identify candidate subjects within the first 24 hours of their admission. Upon identification of a potential subject, study staff will immediately contact the clinical care team and provide them with information regarding the study. A member of the clinical care team who is already known to the patient will gauge the appropriateness of approaching them. If they deem it appropriate, the clinical care team member will approach the patient to determine if they are willing to learn about this study. If the patient agrees, the clinical care team member will authorize the study staff to approach the patient to discuss the study. The described process will take place within 24 hours of admission.

Patients will be consented according to the Partners IRB's Guidelines for Consenting in COVID Research that is More than Minimal Risk. Participants will be enrolled from inpatient floors only. There will be no enrollment of subjects directly from the Emergency Department.

Pretreatment and Baseline Assessments

Baseline Visit

Subjects will be screened within 24 hours of their initial presentation to determine eligibility and provide informed consent for participation in the study.

The following will be performed and documented during screening to assess eligiblity

- Obtain informed consent
- Determine inclusion and exclusion eligibility
- Obtain relevant medical history
- Obtain details of concomitant medications
- Complete physical examination including vital signs (resting blood pressure, pulse, respiratory rate and temperature), body weight, and height
- Obtain nasopharyngeal swabs for COVID-19 quantitative PCR testing
- Collect 30ml research blood for storage
- Collect sputum for storage
- Review of clinical lab results for
    - Chemistry
    - Hematology
    - Coagulation
    - Pregnancy testing in WOCBP
    - Immune biomarkers (IFN- γ, IL-6, IP-10, MCP-1, TNF-α)

  Clinical labs drawn within 24 hours prior to consent (48 hours for pregnancy testing and immune biomarkers) will be used for screening and as baseline. If clinical labs are not available, subject will be deemed a screen failure or consent will be delayed until clinically indicated results become available.

- Request completion of baseline symptom questionnaire

Nasopharyngeal samples will be collected using a standardized method and will be sent to a central laboratory for processing. Both nostrils will be samples.

From the time of obtaining informed consent through the first administration of study drug (Lambda or saline placebo), all serious adverse events (SAEs), as well as any adverse events related to protocol-mandated procedures, will be recorded in the source and on the adverse events case report form (CRF/eCRF). All other untoward medical occurrences observed during the screening period, including exacerbation or changes in medical history are to be captured on the medical history CRF/eCRF.

See Appendix 3: Adverse Events and Toxicity Management for additional details.

After review of inclusion and exclusion criteria to confirm continued eligibility, subjects will move into the baseline portion of the visit and be randomized to study drug or placebo assignment and given a Subject Identification Number via the randomization system. Study drug or placebo will be administered on-site by appropriately credentialed and trailed staff. Upto 30 patients will be consented with a plan of 20 patients randomized to receive Lambda or placeo. A 10 patient cap will be placed on the Lambda treatment group, study enrollment will be halted if this cap is reached.

The following will be performed and documented:

- Review of inclusion/exclusion criteria
- Review of concomitant medications taken since previous visit
- Randomization to study drug or standard of care arm
- Obtain nasopharyngeal swab for COVID-19 PCR testing (pre-dose)
- Administration of first dose of study drug (Lambda) or placebo (saline)

- Collection of research sputum for storage
- Collection of 30ml research blood for storage

Post-treatment Assessments

Days 3, 5, 7 and 14**

The following will be performed and documented:

- Symptom driven physical exam
  - *Clinically performed physical exams may be used in place of research exam when performed same day of visit during inpatient stay*
- Vital signs as recorded by hospital care team
- Obtain nasopharyngeal swabs for COVID-19 quantitative PCR testing
- Request completion of symptom questionnaire
- Record all concomitant medications that the subject has taken since the previous visit
- Record any serious adverse events and all adverse events related to protocol mandated procedures occurring since the Screening visit
- Blood samples for clinical testing (Day 14 only):
  - Chemistry
  - Hematology
  - Coagulation
  - Immune biomarkers (IFN- γ, IL-6, IP-10, MCP-1, TNF-α)
- Collect sputum and 30ml research blood for storage (Day 7 and 14 only)
- Administration of Lambda/placebo injection (Day 7 only)

**Study Days 7 and 14 will have a protocol window of +/- 1 day*

In the event that study subjects are discharged before the end of follow up, they will be requested to present to the RACC for the remaining study time points, where a research nurse will conduct the study visit and obtain swab samples. Subjects will be strongly discouraged from using taxi or rideshare services to return for outpatient visits in the RACC. Parking vouchers (purchased with study funds) will be provided to subjects to cover out of pocket cost of parking.

Upon completion of the Day 14 visit, subjects will be instructed their study participation has ended and will be referred back to their care team for any necessary clinical follow-up, inclusive of additional clinical COVID PCR testing, as needed. Female subjects of childbearing potential will be instructed to self-report a pregnancy that occurs within 30 days of their day 7 dose, but study staff will not directly query subjects regarding pregnancy after Day 14.

Blood draws will be performed by qualified personnel at the request of study staff. Research coordinators will never perform blood draws. Blood samples may be drawn from arterial or central lines or PICC catheters. They will not be drawn from peripheral lines, except at the time of placement of a new peripheral line. Blood samples for research purposes will be drawn alongside clinical samples, wherever possible. Alternatively, lab results obtained close to study time points for clinical purposes may be used, if available.

Unscheduled Visits

Additional unscheduled assessments may be performed at the discretion of the investigator.

Early Termination Visit

In the event a subject terminates the study prior to completion of the 14 day follow up period, the Early Termination (ET) visit should be scheduled as soon as possible. The early termination visit procedures are outlined in Appendix 2 and mirror the day 14 visit but without research archive sample collection.

VII. Adverse Events and Toxicity Management

Definition of Adverse Events, Adverse Reactions and Serious Adverse Events

Adverse Event

An adverse event (AE) is any untoward medical occurrence in a clinical study subject administered a medicinal product, which does not necessarily have a causal relationship with the treatment. An AE can therefore be any unfavorable and/or unintended sign, symptom, or disease temporally associated with the use of a medicinal product, whether or not considered related to the medicinal product. AEs may also include pre- or post-treatment complications that occur as a result of protocol specified procedures, overdose, drug abuse/misuse reports, or occupational exposure. Preexisting events that increase in severity or change in nature during or as a consequence of participation in the clinical study will also be considered AEs.

An AE does not include the following:

- Medical or surgical procedures such as surgery, endoscopy, tooth extraction, and transfusion.
- The condition that led to the procedure may be an adverse event and must be reported
- Pre-existing diseases, conditions, or laboratory abnormalities present or detected before the Screening visit that do not worsen
- Situations where an untoward medical occurrence has not occurred (e.g., hospitalization for
- elective surgery, social and/or convenience admissions)
- Overdose without clinical sequelae
- Any medical condition or clinically significant laboratory abnormality with an onset date before the consent form is signed and not related to a protocol-associated procedure is not an AE. It is considered to be pre-existing and should be documented on the medical history case report form (CRF)

Serious Adverse Event

A **serious adverse event** (SAE) is defined as an event that, at any dose, results in the following:

- Death
- Life-threatening (Note: The term “life-threatening” in the definition of “serious” refers to an event in which the subject was at risk of death at the time of the event; it does not refer to an event that hypothetically might have caused death if it were more severe)
- In-patient hospitalization or prolongation of existing hospitalization
    - Hospitalization for an elective procedure is not considered an SAE for the purposes of this study
- Persistent or significant disability/incapacity
- A congenital anomaly/birth defect

- A medically important event or reaction: such events may not be immediately life-threatening or result in death or hospitalization but may jeopardize the subject or may require intervention to prevent one of the other outcomes constituting SAEs. Medical and scientific judgment must be exercised to determine whether such an event is a reportable under expedited reporting rules.

Examples of medically important events include:
- intensive treatment in an emergency room or at home for allergic bronchospasm
- blood dyscrasias or convulsions that do not result in hospitalization
- development of drug dependency or drug abuse

Clinical Laboratory Abnormalities and Other Abnormal Assessments as Adverse Events or Serious Adverse Events

Laboratory abnormalities without clinical significance are not recorded as AEs or SAEs. However, laboratory abnormalities (e.g., clinical chemistry, hematology) that require medical or surgical intervention or lead to investigational medicinal product (IMP) interruption, modification, or discontinuation must be recorded as an AE, as well as an SAE, if applicable. In addition, laboratory or other abnormal assessments (e.g., vital signs) that are associated with signs and/or symptoms must be recorded as an AE or SAE if they meet the definition of an AE or SAE as described above. If the laboratory abnormality is part of a syndrome, record the syndrome or diagnosis (e.g., anemia), not the laboratory result (i.e., decreased hemoglobin).

Assessment of Adverse Events and Serious Adverse Events

The investigator or qualified sub investigator is responsible for assessing AEs and SAEs for causality and severity, and for final review and confirmation of accuracy of event information and assessments.

Assessment of Causality for Study Drugs and Procedures

The investigator or qualified sub investigator is responsible for assessing the relationship to IMP therapy using clinical judgment describing the event as either unrelated (No) or related (Yes) consistent with the following definitions:
- **No**: Evidence exists that the adverse event has an etiology other than the IMP. For SAEs, an alternative causality must be provided (e.g., pre-existing condition, underlying disease, intercurrent illness, or concomitant medication)

- **Yes**: There is reasonable possibility that the event may have been caused by the investigational medicinal product

It should be emphasized that ineffective treatment should not be considered as causally related in the context of adverse event reporting.

The relationship of an AE or SAE to study procedures (e.g., invasive procedures such as venipuncture or biopsy) should be assessed using the following considerations:
- **No:** Evidence exists that the adverse event has an etiology other than the study procedure

- **Yes:** The adverse event occurred as a result of protocol procedures, (e.g., venipuncture)

Extra consideration will be made regarding fever as an adverse event. Fever is noted as a less common (<10%) potential side effect of Lambda, as well as a common expected symptom of COVID-19 infection. To best distinguish between fever associated with COVID-19 and Lambda treatment emergent or exacerbation of fever, study investigators will adjudicate suspected causality by reviewing time elapsed between Lambda administration and onset/exacerbation of fever. We acknowledge that this distinction cannot be made with complete accuracy.

Assessment of Severity

The severity grading of AEs will be assessed as Grade 1, 2, 3, 4, or 5 according to the Common Terminology Criteria for Adverse Events (CTCAE) Version 4.03, which can be found at http://evs.nci.nih.gov/ftp1/CTCAE/CTCAE_4.03_2010-06-14_QuickReference_8.5x11.pdf.

For AEs associated with laboratory abnormalities, the event should be graded on the basis of the clinical severity in the context of the underlying conditions; this may or may not be in agreement with the grading of the laboratory abnormality.

The distinction between the seriousness and the severity of an adverse event should be noted. Severe is a measure of intensity; thus, a severe reaction is not necessarily a serious reaction. For example, a headache may be severe in intensity, but would not be classified as serious unless it met one of the criteria for serious events listed above.

Reporting of Adverse Events

The Principal Investigator (Dr. Raymond Chung) will be immediately notified of any adverse events. These will be documented in detail in the Adverse Events Tracking Log and submitted each year at the time of continuing review. Adverse events will be reported to the PHRC as described in the PHRC policy on Unanticipated Problems Involving Risks to Subjects or Others.

VIII. Statistical Considerations

Data Analysis

All subjects will be included in the primary intention-to-treat analysis as well as the analyses of secondary outcomes and observed safety events. The primary analysis will compare the proportion of subjects who are PCR negative at post-treatment Day 7 between subjects receiving Lambda vs. standard of care using Fisher's exact test, and the difference between the treatment groups will be estimated using the difference in proportions. Subjects who die during follow up will be considered not to have achieved viral clearance, for the purposes of statistical analysis. Subjects who are lost to follow up will be removed from the analysis, since we consider loss to follow up as being unrelated to our outcome of interest. A key secondary analysis will compare the mean change in quantitative PCR from baseline to Day 7 in the treatment group and placebo group using a two-sample t-test. This analysis will likely have more power to detect a treatment effect compared to the PCR negative analysis since a continuous outcome will be used.

Additional secondary analyses will compare the proportion of subjects who are PCR negative at post-treatment Days 3, 5, and 14 and the mean magnitude of viral load reduction using the same approach at Day 7. In addition, the time to PCR negativity will be compared using a log-rank test, and the difference between the treatment groups will be estimated using a Cox proportional hazards model. To compare

changes in symptom scores over time, a linear mixed model will be used estimate the difference in the change with time between the two treatment groups at each of the post-baseline timepoints. For safety endpoints, the proportion of subjects who experience each type of event will be compared between the Lambda vs. standard of care groups at Day 14 using Fisher's exact test. Additional analysis will compare proportions of subjects who experience progression of disease as reflected by transfer to ICU or intubation, and the length of stay among hospitalized subjects will be compared using the log-rank test. Subjects who die will be analyzed as having a length of stay censored at one day past the last follow-up date. Hence, subjects who die will be considered as having a greater length of stay than subjects who remain alive for the duration of the study. Statistical analyses will be completed using SAS (SAS Institute 2011, Version 9.4, Cary, NC).

Sample size and power calculation:

This is a pilot study to assess the ability of Lambda to increase the proportion of subjects with PCR negativity at Day 7 compared to standard of care (placebo group) as a primary endpoint. We anticipate that no subjects in the standard of care arm will be PCR negative at Day 7, and we anticipate that 50% of subjects in the Lambda arm will be PCR negative at Day 7.

The treatment will be considered for further study if the proportion of subjects who achieve PCR negativity will be 30% higher at Day 7 in the Lambda arm compared to the standard of care arm. This would correspond to 3/10 subjects in the Lambda arm if 0/10 subjects were PCR negative in the standard of care (placebo) arm. Assuming 50% of subjects will be PCR negative, we have a 94% chance of observing 3 or more subjects who are PCR negative at Day 7. This shows that our sample size of 10 subjects per group is sufficient to have a reasonable chance to support further study given the proposed treatment effect. Additionally, demonstration of a reduction in viral load relative to the placebo arm will be factored into decisions to pursue further trials.

IX. References

1. Read SA, Wijaya R, Ramezani-Moghadam M, Tay E, Schibeci S, Liddle C, Lam VWT, Yuen L, Douglas MW, Booth D, George J, Ahlenstiel G. Macrophage Coordination of the Interferon Lambda Immune Response. Front Immunol. 2019 Nov 19; 10:2674.
2. Mordstein M, Neugebauer E, Ditt V, Jessen B, Rieger T, Falcone V, et al. Lambda interferon renders epithelial cells of the respiratory and gastrointestinal tracts resistant to viral infections. J Virol. 2010;84:5670–7.
3. Loutfy MR et al. Interferon alfacon-1 plus corticosteroids in severe acute respiratory syndrome: A preliminary study. JAMA 2003 Dec 24/31; 290:3222-8.

Appendices

Appendix 1: Investigator signature page

Appendix 2: Study procedures table for hospitalized subjects (schedule of events)

Appendix 3: GSI Grading scale for Severity of Adverse Events and Lab Abnormalities

Appendix 4: Pregnancy precautions/definition of Woman of Childbearing potential/contraceptive requirements

Appendix 5: Symptom Questionnaire

Appendix 1: Investigator Signature Page

Investigator Signature Page

MASSACHUSETTS GENERAL HOSPITAL
55 FRUIT STREET
BOSTON, MA 02114

STUDY ACKNOWLEDGEMENT

A Randomized Controlled Trial to Evaluate the Safety and Efficacy of Pegylated Interferon Lambda vs. Standard Supportive Care in Subjects Infected with COVID-19

Protocol Version 1.2 dated 26 APRIL 2020

INVESTIGATOR STATEMENT

I have read the protocol, including all appendices, and I agree that it contains all necessary details for me and my staff to conduct this study as described. I will conduct this study as outlined herein and will make a reasonable effort to complete the study within the time designated.

I will provide all study personnel under my supervision copies of the protocol and access to all information provided by Massachusetts General Hospital. I will discuss this material with them to ensure that they are fully informed about the drugs and the study.

______________________________________	__________________________________
Principal Investigator Name (Printed)	Signature
______________________________________	__________________________________
Date	Site Name/Location

Appendix 2: Study Procedures Table

	On-Study				End of study	
	**Screening/Baseline**	**Day 3**	**Day 5**	**Day 7**	**Day 14** (EOS)	**Early Termination**
Study Visit	*V1*	*V2*	*V3*	*V4*	*V5/EOS*	*ET*
**Assessments**						
Informed Consent	x					
Chemistry *(BMP, AST, ALT, AlkP, tbili, dbili)*	x				x	
Hematology *(CBC w/ diff)*	x				x	
Immune Markers *(IFN- γ, IL-6, IP-10, MCP-1, TNF-α)*	x				x	
Coagulation (PT/INR)	x					
Serum Pregnancy*	x					
Physical examination^	x	x^	x^	x^	x^	x^
Height/Weight	x					
Vital signs *(BP, HR, RR, temp)*	x	x	x	x	x	x
Inclusion/Exclusion criteria confirmation	x					
Randomization	x					
Administration of Lambda/Placebo	x			x		
Nasal Swabs for PCR analysis and storage	x	x	x	x	x	x
Concomitant Medications	x	x	x	x	x	x
Symptom questionnaire	x	x	x	x	x	x
Adverse events	x	x	x	x	x	x
Research sputum for storage	x			x	x	
Research blood for storage (30cc)	x			x	x	

**WOCBP only*

^symptom driven PE

Appendix 3: GIS Grading Scale for Severity of Adverse Events and Laboratory Abnormalities

https://www.fda.gov/media/73679/download

Appendix 4: Pregnancy Precautions and Definitions

The below listed methods meet the requirements for contraception as per the CTFG guidance.

If female, subject must be either postmenopausal defined as:

- Age ≥ 55 years with no menses for 12 or more months without an alternative medical cause.
- Age < 55 years with no menses for 12 or more months without an alternative medical cause AND an FSH level > 40 IU/L.

  OR

- Permanently surgically sterile (bilateral oophorectomy, bilateral salpingectomy or hysterectomy).

Agreeing to practice one effective method of birth control while receiving study drug (as outlined in the subject information and consent form or other subject information documents), starting with Day 1 and for at least 30 days after stopping study drug.

- Bilateral tubal occlusion/ligation.
- Vasectomized partner(s), provided the vasectomized partner has received medical assessment of the surgical success and is the sole sexual partner of the WOCBP trial participant.
- Hormone-releasing Intrauterine device (IUD)
- Hormone-releasing Intrauterine hormone-releasing system (IUS)
- Male or female condom with spermicide (male and female condom must not be used together)
- Diaphragm with spermicide
- Cervical cap with spermicide
- Contraceptive sponge with spermicide
- True abstinence:
  - Refraining from heterosexual intercourse when this is in line with the preferred and usual lifestyle of the subject (periodic abstinence, e.g., calendar, ovulation, symptothermal, post-ovulation methods, “rhythm method,” or pre-ejaculation withdrawal, are not acceptable forms of contraception).
  - Sexually active with female partners only

Appendix 5: Symptom Questionnaire

Symptom questionnaire will be available as a paper tool as well as an electronic tool (RedCap survey). Patients may complete electronically and submit to study team (preferably via RedCap, email alternative option) or have the option to complete via interview and answers will be dictated onto the paper form by a caregiver, member of the study team or member of the clinical team.

MASSACHUSETTS GENERAL HOSPITAL
LIVER CENTER

DAILY SYMPTOM QUESTIONNAIRE

Subject ID:______________ Date:______________ (mm/dd/yyyy) Protocol #: 2020P001083

This survey asks you to record your daily symptoms. This information will help keep track of how you feel and if your symptoms worsen or improve. Thank you for completing this survey!

For each of the questions below, please circle or mark one number per line to indicate your response as it applies to the last 24 hours (1 day).

	Not at all	A little bit	Some-what	Quite a bit	Very much
Muscle pain *(Myalgia)*	◯	◯	◯	◯	◯
Loss of smell *(Anosmia)*	◯	◯	◯	◯	◯
Loss of taste	◯	◯	◯	◯	◯
Cough	◯	◯	◯	◯	◯
Shortness of breath *(Dyspnea)*	◯	◯	◯	◯	◯
Chills	◯	◯	◯	◯	◯
Fever *(Feeling feverish)*	◯	◯	◯	◯	◯
Repeated Shaking with Chills	◯	◯	◯	◯	◯
Headache	◯	◯	◯	◯	◯
Sore throat	◯	◯	◯	◯	◯

Clear Form | Save Form

Please check form for completion - ensure subject ID, date and all questions are answered

Email completed daily symptom questionnaire to: MGHLambdaStudy@partners.org

COVID-SQ version 1.1
Page 1 of 1
