# Supplementary material for: Peginterferon lambda for the treatment of hospitalized patients with mild COVID-19: A pilot phase 2 randomized placebo-controlled trial
Source: Front Med (Lausanne). 2023 Feb 24;10:1095828. doi: 10.3389/fmed.2023.1095828 (PMC10002416; doi:10.3389/fmed.2023.1095828)
Supplement: Supplementary file 2 [file Table_1.pdf]

ID	Group	Age	Gender	Year and month at baseline
1003	Placebo	61	Male	2020 July
1004	Treatment	54	Male	2020 July
1005	Placebo	40	Male	2020 September
1006	Treatment	42	Male	2020 September
1007	Treatment	48	Female	2020 October
1009	Placebo	72	Male	2020 October
1011	Placebo	59	Female	2020 December
1012	Treatment	62	Male	2020 December
1013	Treatment	62	Male	2020 December
1015	Placebo	49	Male	2021 January
1016	Placebo	49	Male	2021 February
1017	Treatment	43	Female	2021 March
1018	Placebo	60	Male	2021 April
1019	Treatment	55	Male	2021 July

Year and month at baseline	Pneumonia at baseline
2020 July	No
2020 July	No
2020 September	No
2020 September	No
2020 October	No
2020 October	No
2020 December	No
2020 December	No
2020 December	No
2021 January	No
2021 February	No
2021 March	No
2021 April	No
2021 July	No

Oxygen therapy through nasal cannula at baseline
No
No
No
No
No
No
No
No
No
No
No
No
No
No
