# Supplementary figures and images for: Peginterferon lambda for the treatment of hospitalized patients with mild COVID-19: A pilot phase 2 randomized placebo-controlled trial
Source: Front Med (Lausanne). 2023 Feb 24;10:1095828. doi: 10.3389/fmed.2023.1095828 (PMC10002416; doi:10.3389/fmed.2023.1095828)

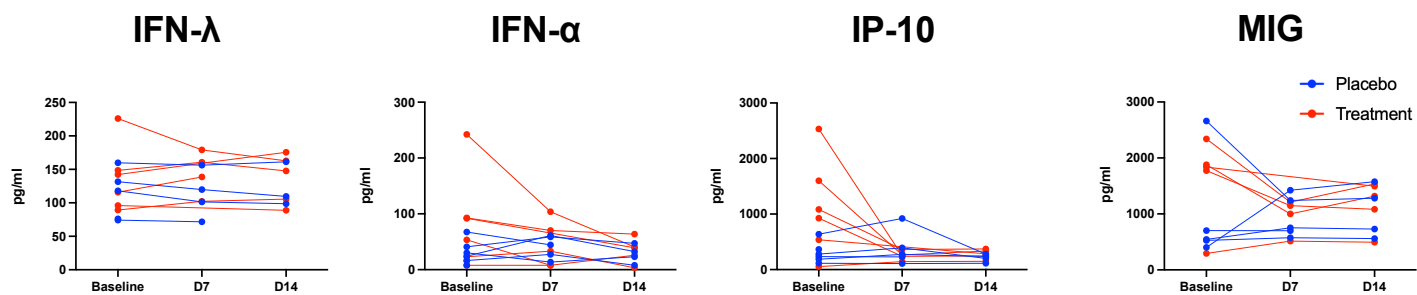

**Supplementary Figure 1. Plasma cytokine levels at baseline, D7, and D14 of each subjects.**

Supplement: Supplementary file 3 [file Image_1.pdf]
